# Attention-Deficit Hyperactivity Disorder (ADHD) Symptoms in Children Adopted from Poland and their Atypical Association Patterns: a Bayesian Approach

**DOI:** 10.1007/s10802-017-0307-4

**Published:** 2017-05-18

**Authors:** Donna A. de Maat, Sandra Knuiman, Catharina H. A. M. Rijk, René A. C. Hoksbergen, Anneloes L. van Baar

**Affiliations:** 1 Utrecht Centre for Child and Adolescent Studies, Utrecht University, Heidelberglaan 1, 3584 CS Utrecht, The Netherlands; 2 Department of Applied Psychology, Thomas More University College of the Catholic University of Leuven, Antwerp, Belgium; 3 Adoption Department, Social and Behavioural Sciences, Utrecht University, Utrecht, The Netherlands

**Keywords:** International adoption, ADHD symptoms, Institutionalization, Early deprivation, Prenatal alcohol exposure

## Abstract

Although high rates of attention-deficit hyperactivity disorder (ADHD) symptoms have been observed among internationally adopted children, research on these symptoms in Polish adoptees is lacking. Therefore, we examined ADHD symptoms in Polish adoptees and their relationship to pre-adoptive risk factors, that is, time in institutional care, early deprivation, and prenatal alcohol exposure. We further compared the association patterns and gender distribution of ADHD symptoms in children adopted from Poland to those reported in the literature for ADHD symptoms in non-adopted children. Dutch adoptive parents of 121 Polish adoptees (52% boys; *M*_age_ = 10.9 years, range 6.2–15.6; *M*_ageadoption_ = 3.0 years, range 0.8–6.9) completed questionnaires regarding ADHD symptoms, pre-adoptive risk factors, attachment problems, conduct problems, and executive functioning deficits. Bayesian evaluation of informative hypotheses showed that Polish adoptees had increased levels of ADHD symptoms, compared to Dutch children in the general population. Time in institutional care, early deprivation, and prenatal alcohol exposure were not associated with ADHD symptoms. ADHD symptoms in Polish adoptees were more strongly associated with attachment problems and executive functioning deficits, but less strongly with conduct problems, compared to ADHD symptoms in non-adoptees. Furthermore, ADHD symptoms were more equally distributed among boys and girls than they are in non-adopted children. The findings indicate that Polish adoptees and their adoptive parents need special attention and support. The dissimilarities between ADHD symptoms in Polish adoptees and non-adoptees might indicate a different underlying causal mechanism, which may have important implications for clinical practice.

Internationally adopted children have shown to be at higher risk for behaviour problems than non-adopted children (e.g., Hawk and McCall 2010; Juffer and Van IJzendoorn 2005). Children adopted from Central Eastern European (CEE) countries experience more behaviour problems in several domains than children adopted from other areas of the world (Gunnar et al. 2007). For instance, these adoptees are more likely to show attention and hyperactivity problems, that is, symptoms characteristic of attention-deficit hyperactivity disorder (ADHD; e.g., Abrines et al. 2012b; Colvert et al. 2008; Hoksbergen et al. 2003; Kreppner et al. 2001; Kumsta et al. 2015). However, research on the development of adoptees from CEE countries has mainly focused on adoptees from Romania and Russia (e.g., Kumsta et al. 2015; Rijk et al. 2010; Robinson et al. 2015; Rutter et al. 2007), while children adopted from Poland have received little attention. More information concerning ADHD symptoms in Polish adoptees is needed, since the majority of these children had experienced multiple pre-adoptive risk factors (Knuiman et al. 2014) that might increase the risk for attention and hyperactivity problems.

Therefore, the current study examined the occurrence of ADHD symptoms in Polish adoptees and their relationship to pre-adoptive risk factors. Additionally, we examined attention deficits, hyperactivity, and impulsivity separately, since these ADHD symptoms constitute essentially different behaviours. In this way, we could explore whether specific characteristics of ADHD occurred and were related to the pre-adoptive risk factors. As previous findings suggested that ADHD symptoms in international adoptees might co-occur with atypical problems, we also compared the association patterns (i.e., the patterns of co- occurring problems) of ADHD symptoms in Polish adoptees to those in non-adopted children. We used a Bayesian approach to overcome the statistical limitations due to small sample size that were encountered in previous adoption studies (e.g., Groza et al. 2008; Knuiman et al. 2014; Sonuga-Barke and Rubia 2008).

This study may increase our understanding of behaviour problems in international adoptees with multiple early risk factors as well as their dissimilarities to behaviour problems often found in the general population of children. This knowledge may help adoptive parents and professionals working with adoptees to offer the most appropriate care. In addition, the current study adds to the literature on adoptees from CEE countries by taking into account multiple risk factors, whereas previous studies mainly focused on children’s adjustment after institutionalization (e.g., Merz and McCall 2010; Rutter et al. 2007; Stevens et al. 2008). That is, previous studies exclusively included children that had stayed in institutional care before adoption. The Polish adoptees in the current sample, however, came from diverse backgrounds. Due to the diversity of this sample, we could consider the role of diverse pre- adoptive risk factors in the presence of later ADHD symptoms among CEE adoptees.

## Pre-Adoptive Risk Factors for ADHD Symptoms

Different pre-adoptive risk factors have been proposed that may explain the high prevalence of ADHD symptoms in internationally adopted children. First of all, early institutionalization has been consistently related to increased levels of ADHD symptoms among adopted children (Gunnar et al. 2007; Hawk and McCall 2010; MacLean 2003). Based on these findings, Rutter et al. (2001) suggested that ADHD symptoms in international adoptees constitute a specific response to early institutional deprivation. That is, children in institutions often have fewer opportunities to experience and engage in warm and responsive caregiver-child interactions, because of high child/caregiver ratios and changing caregivers (Hawk and McCall 2010). According to attachment theory (Bowlby 1982), such deficits in early caregiver-child interactions may hinder the formation of secure attachment relationships and impair the child’s self-regulation abilities: Children with poor self- regulation abilities are known to be at risk for attention, hyperactivity, and impulsivity problems (Walcott and Landau 2004). A neuropsychological explanation may be that lacking consistent and responsive interactions early in life can disturb the development of brain regions, including areas in the prefrontal cortex important for attention and the processing of social information. Impairment of these brain areas can, in turn, result in symptoms characteristic of ADHD (Roy et al. 2004). Indeed, Romanian adoptees who had spent more time in severely depriving institutions showed more ADHD symptoms (Kreppner et al. 2001; Rutter et al. 2007), supporting the idea that these symptoms may be partially caused by institutional deprivation.

Previous studies among adopted children mainly focused on deprivation in institutional settings, while early deprivation in general has received less attention. Nevertheless, international adoptees come from diverse backgrounds and may have experienced early deprivation in diverse environments. Apart from having resided in institutions, some of these children had remained in a hospital for weeks before adoption, sometimes receiving only minimal attention from caregivers, whereas others had been deprived by their biological parents (Groza and Ryan 2002; Hawk and McCall 2010). There is evidence among non-adopted children that early deprivation by a parent or caregiver increases the risk for several behaviour problems, amongst which ADHD symptoms (Maguire et al. 2015). Likewise, international adoptees that had experienced deprivation prior to adoption displayed increased levels of internalizing and externalizing problems (Van der Vegt et al. 2009). It is, however, not yet clearly understood whether early deprivation in any environment contributes to the increased levels of ADHD symptoms in international adoptees.

Another pre-adoptive risk factor that might be related to the increased ADHD symptoms in international adoptees is prenatal alcohol exposure. Maternal alcohol consumption during pregnancy may have long-lasting adverse effects, causing physical, neurological, and behavioural damage (Mattson et al. 2011). It is known that prenatal alcohol exposure may directly affect the developing brain and cause impairment in various neuropsychological functions, including attention and activity levels (Mattson et al. 2011). Indeed, non-adopted children prenatally exposed to alcohol were likely to display attention deficits, hyperactivity, and impulsivity (Fryer et al. 2007). Despite these findings, no study has investigated the contribution of prenatal alcohol exposure to the development of ADHD symptoms in international adoptees.

## Children Adopted from Poland

Children from Poland constitute a substantial group of international adoptees within Europe (Selman 2010). In the Netherlands, they comprise the largest group of children adopted from CEE countries between 1999 and 2006 (Hoksbergen 2012). It is known that institutionalization, early deprivation, and prenatal alcohol exposure are highly prevalent among adoptees from CEE countries (e.g., Gunnar et al. 2007; Landgren et al. 2010; Miller et al. 2009). Likewise, the majority (83%) of children adopted from Poland had stayed in institutional care prior to adoption, for example in orphanages and baby homes. These children had stayed in an institution for 2 years on average (Knuiman et al. 2014). In addition, most adoptees from Poland (65%) had a history of early deprivation. Deprivation by the biological parents was one of the main reasons for their out-of-home placement (Knuiman et al. 2015b). Children adopted from Poland were also found to be at high risk for prenatal alcohol exposure: About one third (31%) of these children were diagnosed with fetal alcohol spectrum disorders (FASD), referring to a range of problems caused by exposure to alcohol during pregnancy (Knuiman et al. 2015a).

However, children adopted from Poland may have experienced different pre-adoption environments than adoptees from other CEE countries, such as Romania. Previous studies among Romanian adoptees were all conducted among children that had experienced severe deprivation in institutional care. The conditions in institutions in Poland, although also characterized by lack of social-emotional support and limited nurturing physical contact (Supreme Audit Office 2008), might be less severely depriving than those experienced by children in Romanian institutions. For instance, the basic needs of the children in Polish institutions, such as food, clothing and medical care, are provided for. Furthermore, Polish adoptees had been exposed to various other risk factors apart from institutionalization, like prenatal alcohol exposure, parental abuse, and being adopted at older age (18 months or older). This co-occurrence of multiple early risk factors makes this a particularly vulnerable group for developing ADHD symptoms. Although the majority of Polish adoptees showed increased problem behaviour (Knuiman et al. 2014), research on ADHD symptoms specifically in this group of adoptees is lacking. A previous study among Polish adoptees found no relationship between pre-adoptive risk factors and general behaviour problems. This result, however, could be due to statistical limitations (Knuiman et al. 2014). Therefore, the current study focused on ADHD symptoms in Polish adoptees using an alternative, Bayesian approach.

## Atypical Association Patterns of ADHD Symptoms in Internationally Adopted Children

The association patterns of ADHD symptoms in adopted children might be somewhat different from those of ADHD symptoms in non-adopted children, possibly due to a different pattern of causation. That is, evidence suggests that ADHD symptoms in international adoptees are influenced by early environmental experiences, whereas ADHD symptoms in non-adopted children are considered to be strongly influenced by genetic factors, with environmental factors playing a subsidiary role in the aetiology (Taylor and Rogers 2005). Although the exact underlying mechanism for the development of attention and hyperactivity problems in international adoptees is unknown, earlier findings indeed point to atypical association patterns of ADHD symptoms in these adoptees: Among Romanian adoptees, ADHD symptoms tended to co-occur with attachment problems and executive functioning deficits (e.g., Rutter et al. 2001; Rutter et al. 2007; Stevens et al. 2008), whereas ADHD symptoms have been consistently associated with conduct problems among non-adopted children (Jensen and Steinhausen 2015; Scholtens et al. 2014; Thorell et al. 2012). A comparison study showed that Romanian adoptees with increased ADHD symptoms displayed less conduct problems and more executive functioning deficits compared to non-adopted children with ADHD (Sonuga-Barke and Rubia 2008). Yet, other findings suggest that ADHD symptoms in adoptees might also be associated with conduct problems (Stevens et al. 2008). This highlights the need for more research into the problems that tend to co-occur with ADHD symptoms in international adoptees.

The gender distribution of ADHD symptoms in adoptees differs from that of ADHD symptoms in non-adopted children as well. In adoptees, ADHD symptoms were found to equally affect boys and girls (Abrines et al. 2012b; Kreppner et al. 2001; Kumsta et al. 2015), whereas ADHD in non-adopted children is much more common in boys than in girls, with ratios of boys to girls reported to be between 2:1 and 9:1 (Ramtekkar et al. 2009; Skogli et al. 2013; Thorell et al. 2012). Although some studies reported higher levels of ADHD symptoms in adopted boys than girls, gender ratios ranged from 1.6:1 to 2:1 (Abrines et al. 2012a; Stevens et al. 2008), pointing to a less biased gender distribution of ADHD symptoms in adopted children than in non-adopted children.

Unfortunately, the few studies that investigated the association patterns and gender distribution of ADHD symptoms were all conducted among Romanian adoptees that had experienced severe deprivation in institutional care. It is therefore unknown whether this atypical presentation of ADHD symptoms can also be found in international adoptees from a different background. Therefore, the current study investigated the association patterns and gender distribution of ADHD symptoms in a group of Polish adoptees, who come from diverse backgrounds and had experienced multiple early risk factors. It is important to better understand the dissimilarities to ADHD symptoms in non-adopted children, since atypical association patterns might have implications for the clinical treatment of international adoptees with ADHD.

## The Current Study

To increase our knowledge about ADHD symptoms in Polish adoptees, the current study aimed to (1) examine the occurrence of ADHD symptoms in Dutch children adopted from Poland and their relationship to pre-adoptive risk factors. Based on previous research, we hypothesized that Polish adoptees would show increased levels of ADHD symptoms, compared to Dutch children in the general population. We more specifically expected that Polish adoptees would display higher levels of attention deficits, hyperactivity, and impulsivity. In addition, we hypothesized that more time in institutional care, early deprivation, and prenatal alcohol exposure would predict higher levels of ADHD symptoms as well as higher levels of attention deficits, hyperactivity, and impulsivity. A second aim of this study was to (2) compare the association patterns and gender distribution of ADHD symptoms in children adopted from Poland to those reported in the literature for ADHD symptoms in non-adopted children. We expected that ADHD symptoms in Polish adoptees would be more strongly associated with attachment problems and executive functioning deficits, but less strongly with conduct problems, than ADHD symptoms are in non-adopted children. Finally, we expected that ADHD symptoms in Polish adoptees would be more equally distributed among boys and girls than these symptoms are in non-adopted children.

## Method

### Participants

The current study was part of a larger project on the development of Polish adoptees in the Netherlands (Knuiman 2015). The eligible population consisted of all 181 children adopted from Poland to the Netherlands between January 1999 and December 2006. To reduce burden among participants, data was collected across two waves with a 2-year interval. At wave 1, the adoptive parents of 133 Polish children completed questionnaires (133/181 = 74% response rate). At wave 2, 91% of these adoptive parents agreed to participate again, resulting in a sample of 121 children (121/181 = 67% response rate) between 6.2 and 15.6 years old (*M* = 10.9 years, *SD* = 2.7; 52% boys). The children were adopted by 71 Dutch couples, who adopted 1 to 4 Polish children (*M* = 1.6 children, *SD* = 0.8). Age at adoption ranged between 0.8 and 6.9 years (*M* = 3.0 years, *SD* = 1.6). Questionnaires were completed by the adoptive mothers (73%), adoptive fathers (8%) or both (19%).

Attrition analyses were performed to test whether the children that only participated in wave 1 differed from the children that participated in both wave 1 and 2 on any of the research variables. Results of univariate analyses of variance (ANOVA) showed there were no significant differences between the two groups concerning age, time in institutional care, and conduct problems. Furthermore, results of χ_2_ goodness-of-fit analyses showed that the two groups did not differ regarding gender and early deprivation.

### Procedure

Dutch adoptive parents of all children adopted from Poland between January 1999 and December 2006 (*n* = 181) received an invitation letter to participate in the study. Letters were sent to the parents by the collaborating adoption agency. Parents that did not respond to our invitation within 2 months, received a reminding letter. After agreeing to participate, parents were requested to complete the questionnaires. All procedures performed in this study were in accordance with the ethical standards of Utrecht University, the Netherlands.

### Measures

#### Demographics and Pre-Adoptive Risk Factors

At wave 1, adoptive parents reported on children’s gender, date of birth, and date of adoption. Pre-adoptive risk factors were also reported by adoptive parents, including the occurrence of institutionalization (*yes, no, unknown*), the time children had spent in institutional care (in months), the occurrence of early deprivation (*severely, moderately, no, unknown*), and the occurrence of prenatal alcohol exposure (*yes, no, unknown*).

#### ADHD Symptoms

The ADHD-questionnaire [ADHD-vragenlijst] (AVL; Scholte and Van der Ploeg 2005) was used to measure ADHD symptoms at wave 1 or 2. The AVL consists of three subscales each containing six items: attention deficit (e.g., is easily distracted), hyperactivity (e.g., talks continuously), and impulsivity (e.g., has difficulty to wait his/her turn). Adoptive parents were asked to indicate to what degree the described actions and characteristics applied to their child in the past 6 months. Answers were given on a 5-point Likert type scale (0 = *the child does not act in this way* to 4 = *the child acts in this way very often [every day]*). An ADHD total score was computed by summing the scores on the three subscales, with a higher score implying more ADHD symptoms. Raw AVL scores were transformed into decile scores, which were derived from the Dutch AVL norm group (Scholte and Van der Ploeg 2005). Scores in decile X were in the clinical range (95–100%) or borderline range (90–94%). The Dutch AVL norm group, consisting of 2536 children from the general population between 4 and 18 years old (*M* = 10.1 years, *SD* = 3.2) was used for comparison. The Dutch Testing Committee judged the validity and reliability of the AVL total scale and subscales as good, except for the criterion validity and norms, which are considered acceptable (Evers et al. 2004). In the current sample, the attention deficit subscale of the AVL correlated positively with the attention subscale of the CBCL/4–18 (*r* = 0.64, *p* < 0.001), supporting the construct validity of this questionnaire. The internal consistency was good in this sample, with Cronbach’s alpha being 0.94 for the total scale and 0.86–0.88 for the subscales.

#### Attachment Problems

To measure attachment problems, parents completed the Global Indicationlist Attachment [Globale Indicatielijst Hechting] (GIH; Polderman 2009) at wave 2. This questionnaire consists of 36 items (e.g., Does your child react extremely anxious to strangers) assessing attachment problems, mainly attachment insecurity. Parents were asked to indicate whether their child displayed certain behaviour (0 = *no,* 1 = *sometimes,* 2 = *yes*). A total score for attachment problems was computed by summing the item scores, with a higher score implying more attachment problems. The internal consistency and validity of the GIH are found to be good (Freriks 2002). In the current sample, the internal consistency was good, with Cronbach’s alpha being 0.75.

#### Conduct Problems

The Dutch version of the Child Behaviour Checklist ([CBCL/4–18]; Achenbach 1991; Verhulst et al. 1996) was used to measure conduct problems at wave 1. We computed a conduct problem score based on the DSM-oriented conduct problems scale of the revised CBCL/6–18 version (Achenbach and Rescorla 2001).

The items on this scale match symptoms of conduct disorder as described in the DSM-IV (e.g., cruel to animals; American Psychiatric Association 2000). Adoptive parents were asked to indicate to what degree the described actions and characteristics applied to their child in the past 6 months. Answers were given on a 3-point scale (0 = *not true,* 1 = *somewhat or sometimes true,* 2 = *very true or often true*). As described by Achenbach and Rescorla (2001), a total conduct problem score was computed by summing the scores on the 16 relevant items (items 15, 16, 21, 26, 37, 39, 43, 57, 67, 72, 81, 82, 90, 97, 101, 106). Higher scores implied more conduct problems. The reliability and validity of the conduct problem scale is found to be good for the revised CBCL/6–18 version (Nakamura et al. 2009), which correlates highly with the CBCL/4–18 version used in the current study (Achenbach and Rescorla 2001). In the current sample, internal consistency was good, with Cronbach’s alpha being 0.83.

#### Executive Functioning Deficits

To measure executive functioning deficits, the Dutch version of the Behaviour Rating Inventory of Executive Function ([BRIEF]; Gioia et al. 2000; Smidts and Huizinga 2009) was used at wave 2. This questionnaire consists of 75 items and eight subscales: inhibit (10 items), shift (8 items), emotional control (10 items), initiate (8 items), working memory (10 items), plan/organise (12 items), organisation of materials (6 items), and monitor (8 items). Adoptive parents were asked to rate the degree to which their children displayed certain behaviour (e.g., Forgets what he/she was doing; 1 = *never,* 2 = *sometimes,* 3 = *often*). A global executive composite score was computed by summing the scores on the eight subscales, with a higher score implying more deficits in executive functioning. The internal consistency and construct validity of the Dutch BRIEF are reported to be good (Huizinga and Smidts 2011). In the current sample, internal consistency was good, with Cronbach’s alpha being 0.97 for the global executive composite score and ranging from 0.86 (organisation of materials) to 0.93 (working memory) for the subscales.

### Statistical Analyses

#### Bayesian Evaluation of Informative Hypotheses

To directly evaluate our hypotheses of interest, we analysed the data using a Bayesian approach (Van de Schoot et al. 2014). We performed Bayesian evaluation of informative hypotheses, which are hypotheses that represent explicit expectations of the researcher based on empirical literature (Hoijtink 2011). This approach enabled us to overcome the statistical limitations related to small sample size often encountered in previous adoption studies (e.g., Groza et al. 2008; Knuiman et al. 2014; Sonuga-Barke and Rubia 2008). We used the data to directly evaluate our specific hypotheses, which is more straightforward than testing several null hypotheses that are not directly related to the hypotheses of interest (Van de Schoot et al. 2014). Another advantage of using informative hypotheses was that we could include previous empirical findings in our hypotheses (Kluytmans et al. 2012). By including background knowledge in the analyses, we could directly build upon previous research on ADHD symptoms in both adopted and non-adopted children.

We formulated informative hypotheses based on the results of a systematic literature review conducted in Scopus and PsycINFO (see Table 1). Regarding the levels of ADHD symptoms, it was expected that the mean score of Polish adoptees on ADHD symptoms would be higher than 14.80, which is the mean score of the Dutch AVL norm group (Scholte and Van der Ploeg 2005). Hypotheses concerning the AVL subscales were also based on the mean scores of the Dutch AVL norm group. It was expected that the mean score of Polish adoptees would be higher than 4.70 for attention deficits, higher than 5.30 for hyperactivity, and higher than 4.50 for impulsivity. To examine whether pre-adoptive risk factors predicted the level of ADHD symptoms, a multiple regression analysis was performed. It was expected that the standardized regression coefficients of time in institutional care, early deprivation, and prenatal alcohol exposure would be larger than 0 (e.g., Colvert et al. 2008; Fryer et al. 2007; Hawk and McCall 2010; Rutter et al. 2007). Concerning the AVL subscales, standardized regression coefficients larger than 0 were expected as well.

**Table 1:** Informative hypotheses containing explicit expectations about coefficients

	Hypothesis 1	Hypothesis 2	Hypothesis 3
Levels of ADHD symptoms			
ADHD symptoms	μ > 14.80	μ = 14.80	μ < 14.80
Attention deficits	μ > 4.70	μ = 4.70	μ < 4.70
Hyperactivity	μ > 5.30	μ = 5.30	μ < 5.30
Impulsivity	μ > 4.50	μ = 4.50	μ < 4.50
Predictors of ADHD symptoms			
ADHD symptoms	β_1_ > 0, β_2_ > 0, β_3_ > 0	β_1_ = 0, β_2_ = 0, β_3_ = 0	-
Attention deficits	β_1_ > 0, β_2_ > 0, β_3_ > 0	β_1_ = 0, β_2_ = 0, β_3_ = 0	-
Hyperactivity	β_1_ > 0, β_2_ > 0, β_3_ > 0	β_1_ = 0, β_2_ = 0, β_3_ = 0	-
Impulsivity	β_1_ > 0, β_2_ > 0, β_3_ > 0	β_1_ = 0, β_2_ = 0, β_3_ = 0	-
Association patterns			
Attachment problems	*r*_attachment_ > 0.33	*r*_attachment_ = 0.33	*r*_attachment_ < 0.33
Conduct problems	*r*_conduct_ < 0.68	*r*_conduct_ = 0.68	*r*_conduct_ > 0.68
Executive functioning deficits	*r*_executive_ > 0.33	*r*_executive_ = 0.33	*r*_executive_ < 0.33
Gender distribution	β_gender_ < 0.36	β_gender_ = 0.36	β_gender_ > 0.36

β_1_ = β for time in institution; β_2_ = β for early deprivation; β_3_ = β for prenatal alcohol exposure

The informative hypotheses regarding the association patterns of ADHD symptoms were based on studies that reported the parameters (i.e., correlation and regression coefficients) in which we were interested. Additionally, we used four inclusion criteria to select the studies. First of all, we limited our literature review to studies among children in the general (i.e., non-clinical) population, as one of our aims was to compare ADHD symptoms and their association patterns in Polish adoptees to those seen in children in the general Dutch population. Second, we included studies with a relatively large sample size (*n* > 50), to decrease the probability that the results were obtained by chance. Third, studies with an adequate methodological design (i.e., adequate psychometric quality of the instruments) were included. Fourth, we selected studies that were published recently (2005 or later). Based on these criteria, a total of 17 studies were excluded.

To examine the association between ADHD symptoms and co-occurring problems, correlational analyses were performed. Concerning attachment problems, we expected that the correlation coefficient between ADHD symptoms and attachment problems would be larger than 0.33, based on Pinto et al. (2006) and Thorell et al. (2012). These studies provided correlations between ADHD symptoms and attachment problems among children in the normal population, ranging from 0.23 to 0.40 (*M*_correlation_ = 0.33).

We expected that the correlation coefficient between ADHD symptoms and conduct problems would be smaller than 0.68, based on Scholtens et al. (2014) and Thorell et al. (2012). To our knowledge, these were the most recent studies reporting correlations between ADHD symptoms and conduct problems among children in the normal population: correlations were 0.74 and 0.63, respectively (*M*_correlation_ = 0.68). We expected that the correlation coefficient between ADHD symptoms and executive functioning deficits would be larger than 0.33, based on Scholtens et al. (2014) and Thorell et al. (2012). To our knowledge, these were the most recent studies reporting correlations between ADHD symptoms and different executive functioning components among children in the normal population: correlations ranged from 0.25 to 0.41 (*M*_correlation_ = 0.33). To examine the gender distribution of ADHD symptoms, a linear regression analysis was performed with gender as predictor (girls = 1; boys = 2). We expected that the standardized regression coefficient of gender would be smaller than 0.36, based on Ramtekkar et al. (2009) and Thorell et al. (2012). Both studies reported a standardized regression coefficient for gender on ADHD symptoms of 0.36 among children in the normal population. The finding of Ramtekkar et al. (2009) was based on a large community-based sample of children (*n* = 2704).

As can be seen in Table 1, all informative hypotheses were tested against alternative hypotheses. For instance, the hypothesis concerning the levels of ADHD symptoms (μ > 14.80) was tested against the alternative hypotheses assuming that the mean score of Polish adoptees would be equal to 14.80 (μ = 14.80) or smaller than 14.80 (μ < 14.80).

Subsequently, we entered each set of informative hypotheses (e.g., μ > 14.80, μ = 14.80, and μ < 14.80) in the software program BIEMS (Mulder et al. 2012). Default unconstrained priors were used. To evaluate the informative hypotheses, BIEMS provides a Bayes Factor (BF). The BF refers to the amount of support in the data for each of the entered hypotheses, compared to a hypothesis without constraints on the coefficients: A BF larger than 1 shows support for a specific hypothesis, while a BF smaller than 1 implies support for its alternative unconstrained hypothesis (Klugkist et al. 2005). For each set of hypotheses, we selected the hypothesis that received most support from the data by dividing the BFs of the different hypotheses by each other.

#### Analysis Plan

To examine the level and predictors of ADHD symptoms, we first conducted the analyses with ADHD symptoms as the dependent variable. After that, we performed separate analyses with the subscales attention deficits, hyperactivity, and impulsivity as dependent variables. To control for dependency in the data (children nested within families), additional analyses were performed with one child randomly selected per family (*n* = 71). The intraclass correlation coefficient was 0.16, indicating that 16% of the total variance in ADHD symptoms could be attributed to differences between families. The analyses including one child randomly selected per family yielded the same conclusions as the analyses including the total sample. Therefore, we decided to include the total sample of children in all analyses.

Missing item values on the questionnaires were imputed by the individual’s average on the corresponding (sub)scale. One case was excluded from all analyses because of missing data on the AVL. In total, 62 cases were excluded from the analyses of pre-adoptive risk factors, because information was unknown to parents or missing (not missing at random). Results of ANOVA showed that the children that were excluded from the analyses of pre- adoptive risk factors did not differ from those included regarding ADHD symptoms. Seven cases were excluded from the analyses of conduct problems, because their age fell below the age range of the CBCL or more than 10% of the data on the CBCL was missing. One case was deleted from the analysis of executive functioning deficits, due to inconsistent scores on the BRIEF.

## Results

### Descriptive Statistics

Table 2 shows the descriptive statistics of ADHD symptoms, pre-adoptive risk factors, and associated problems of the Polish adoptees. Compared to the norm group, the percentage of Polish adoptees scoring in the clinical or borderline range for ADHD symptoms was high:

**Table 2:** Descriptive statistics of ADHD symptoms, pre-adoptive risk factors, and associated problems

Variable	*n*	*M (SD)*	%
ADHD symptoms			
ADHD total score	120	26.46 (17.36)	
Attention deficits subscale	120	8.97 (6.51)	
Hyperactivity subscale	120	9.21 (6.81)	
Impulsivity subscale	120	8.28 (5.86)	
Pre-adoptive risk factors^ab^			
Institutionalization	120		
Yes		23.11 (10.42)^c^	82.5
No			17.5
Unknown			-
Early deprivation	118		
Severely			55.1
Moderately			11.9
No			19.5
Unknown			13.6
Prenatal alcohol exposure	121		
Yes			54.5
No			17.4
Unknown			28.1
Associated problems			
Attachment problems	121	23.86 (12.51)	
Conduct problems	113	2.77 (3.68)	
Executive functioning deficits	119	139.52 (31.52)	

^a^ *n* represents the total sample minus the children for whom information on the pre-adoptive risk factor was missing

^b^Two children were reported as not being exposed to any of the three pre-adoptive risk factors

^c^Time in institutional care measured in months.

In total, 40.0% of the Polish adoptees scored in the clinical (25.8%) or borderline range (14.2%), compared to 10.0% of the children in the norm group that scored in the clinical (5.0%) or borderline range (5.0%). The correlations between the three pre-adoptive risk factors were very weak or weak and did not point to multicollinearity. Since children’s age at adoption and at time of study were not related to ADHD symptom levels, these variables were not included in the remaining analyses.

### Levels of ADHD Symptoms

Results of the Bayesian evaluation of informative hypotheses for the level of ADHD symptoms are displayed in Table 3. It was found that hypothesis 1, assuming that Polish adoptees would score higher than 14.80 on ADHD symptoms, received most support in the data. Hypothesis 1 was about 200 times more likely than hypothesis 2 and hypothesis 3 (BF_1vs2_ > 201.00; BF_1vs3_ > 201.00). Similar results were found when testing the hypotheses separately for the subscales attention deficits, hyperactivity, and impulsivity, indicating that Polish adoptees scored higher than the Dutch norm group on all subscales.

**Table 3:** Results of Bayesian evaluation of informative hypotheses for the levels of ADHD symptoms

	Hypothesis 1	Hypothesis 2	Hypothesis 3
ADHD symptoms	**μ > 14.80**	μ = 14.80	μ < 14.80
BF	2.01	<0.01	< 0.01
Attention deficits	**μ > 4.70**	μ = 4.70	μ < 4.70
BF	2.00	< 0.01	< 0.01
Hyperactivity	**μ > 5.30**	μ = 5.30	μ < 5.30
BF	1.99	< 0.01	< 0.01
Impulsivity	**μ > 4.50**	μ = 4.50	μ < 4.50
BF	2.00	< 0.01	< 0.01

Hypotheses that received most support in the data are shown in boldface

*BF* Bayes Factor of current versus unconstrained model

### Predictors of ADHD Symptoms

Results of the Bayesian evaluation of informative hypotheses concerning the predictors of ADHD symptoms are displayed in Table 4. It was found that hypothesis 2, assuming no effects of time in institutional care, early deprivation, and prenatal alcohol exposure on ADHD symptoms, received most support in the data. Hypothesis 2 was about 183 times more likely than hypothesis 1, which assumed that pre-adoptive risk factors would be positively related to ADHD symptoms (BF_2vs1_ = 183.42). Posterior standardized regression coefficients of the predictors were close to zero (β_1_ = −0.04, *SD* = 0.02; β_2_ = −0.06, *SD* = 0.03; β_3_ = 0.10, *SD* = 0.03).^1^ Similar results were found when testing the hypotheses separately for the subscales attention deficits, hyperactivity, and impulsivity, indicating no effect of pre- adoptive risk factors on each of the subscales. Furthermore, additional analyses including only one pre-adoptive risk factor per analysis revealed that none of the pre-adoptive risk factors was associated with ADHD symptoms individually.

**Table 4:** Results of Bayesian evaluation of informative hypotheses for the predictors of ADHD symptoms

	Hypothesis 1	Hypothesis 2
ADHD symptoms	β_1_ > 0, β_2_ > 0, β_3_ > 0	**β**_**1**_ **= 0, β**_**2**_ **= 0, β**_**3**_ **= 0**
BF	0.48	88.04
Attention deficits	β_1_ > 0, β_2_ > 0, β_3_ > 0	**β**_**1**_ **= 0, β**_**2**_ **= 0, β**_**3**_ **= 0**
BF	0.23	12.42
Hyperactivity	β_1_ > 0, β_2_ > 0, β_3_ > 0	**β**_**1**_ **= 0, β**_**2**_ **= 0, β**_**3**_ **= 0**
BF	0.18	69.65
Impulsivity	β_1_ > 0, β_2_ > 0, β_3_ > 0	**β**_**1**_ **= 0, β**_**2**_ **= 0, β**_**3**_ **= 0**
BF	0.56	97.14

Hypotheses that received most support in the data are shown in boldface. β_1_ = β for time in institution; β_2_ = β for early deprivation; β_3_ = β for prenatal alcohol exposure

*BF* Bayes Factor of current versus unconstrained model

### Association Patterns of ADHD Symptoms

Results of the Bayesian evaluation of informative hypotheses concerning the association patterns of ADHD symptoms are displayed in Table 5. It was found that hypothesis 1, assuming that the correlation between ADHD symptoms and attachment problems in Polish adoptees was larger than 0.33, received most support in the data. Hypothesis 1 was about 3 times and 71 times more likely than hypothesis 2 and 3, respectively (BF_1vs2_ = 3.08; BF_1vs3_ = 71.20). As can be seen in the table, the posterior correlation coefficient was estimated at 0.51. Concerning conduct problems, it was found that hypothesis 1, assuming that the correlation between ADHD symptoms and conduct problems in Polish adoptees was smaller than 0.68, received most support in the data. Hypothesis 1 was about 18 times and 627 times more likely than hypothesis 2 and 3, respectively (BF_1vs2_ = 18.09; BF_1vs3_ = 627.05). For executive functioning deficits, it was found that hypothesis 1, assuming the correlation between ADHD symptoms and executive functioning deficits in Polish adoptees was larger than 0.33, received most support in the data. Hypothesis 1 was about 6800 times and more than 1,000,000 times more likely than hypothesis 2 and 3, respectively (BF_1vs2_ = 6770.48; BF_1vs3_ > 1,000,000).

**Table 5:** Results of Bayesian evaluation of informative hypotheses for the association patterns of ADHD symptoms

	Hypothesis 1	Hypothesis 2	Hypothesis 3	Posterior estimation (*SD*)^a^
Attachment problems	***r*** **> 0.33**	*r* = 0.33	*r* < 0.33	*r* = 0.51 (0.01)
BF	1.97	0.64	0.01	
Conduct problems	***r*** **< 0.68**	*r* = 0.68	*r* > 0.68	*r* = 0.42 (0.01)
BF	1.99	0.11	< 0.01	
Executive functioning deficits	***r*** **>** 0**.33**	*r* = 0.33	*r* < 0.33	*r* = 0.71 (0.01)
BF	2.00	< 0.01	< 0.01	

Hypotheses that received most support in the data are shown in boldface

*BF* Bayes Factor of current versus unconstrained model

^a^Posterior estimations refer to the Bayesian equivalents of *r* and are based on a compromise between prior information and the current data

### Gender Distribution of ADHD Symptoms

Results of the Bayesian evaluation of informative hypotheses concerning the gender distribution of ADHD symptoms are displayed in Table 6. It was found that hypothesis 1, assuming that the regression coefficient of gender was smaller than 0.36, received most support in the data. Hypothesis 1 was about 2400 times and more than 1,000,000 times more likely than hypothesis 2 and 3, respectively (BF_1vs2_ = 2375.89; BF_1vs3_ > 1,000,000). Similar results were found when testing the hypotheses separately for the subscales attention deficits, hyperactivity, and impulsivity, indicating the regression coefficient of gender was smaller than 0.36 for all subscales. In line with this finding, boys (*M* = 28.46, *SD* = 18.36) scored only slightly higher than girls (*M* = 24.33, *SD* = 16.12) on ADHD symptoms.

**Table 6:** Results of Bayesian evaluation of informative hypotheses for the gender distribution of ADHD symptoms

	Hypothesis 1	Hypothesis 2	Hypothesis 3	Posterior estimation (*SD*)^a^
ADHD symptoms	**β**_**gender**_ **< 0.36**	β_gender_ = 0.36	β_gender_ > 0.36	β_gender_ = 0.11 (0.01)
BF	2.00	< 0.01	< 0.01	
Attention deficits	**β**_**gender**_ **< 0.36**	β_gender_ = 0.36	β_gender_ > 0.36	β_gender_ = −0.00 (0.01)
BF	1.99	0.01	< 0.01	
Hyperactivity	**β**_**gender**_ **< 0.36**	β_gender_ = 0.36	β_gender_ > 0.36	β_gender_ = 0.08 (0.01)
BF	2.00	< 0.01	< 0.01	
Impulsivity	**β**_**gender**_ **< 0.36**	β_gender_ = 0.36	β_gender_ > 0.36	β_gender_ = 0.04 (0.01)
BF	2.00	< 0.01	< 0.01	

Hypotheses that received most support in the data are shown in boldface

*BF* Bayes Factor of current versus unconstrained model

^a^ Posterior estimations refer to the Bayesian equivalents of β and are based on a compromise between prior information and the current data

## Discussion

The current study aimed to examine the occurrence of ADHD symptoms in Dutch children adopted from Poland and their relationship to pre-adoptive risk factors. As expected, children adopted from Poland showed increased levels of ADHD symptoms, compared to a Dutch norm group of children in the general population. Compared to this norm group, Polish adoptees were four times more likely to have ADHD symptoms at a clinical or borderline level. These results are in line with previous research indicating that children adopted from other CEE countries are more likely to show attention and hyperactivity problems, in comparison to non-adopted children (e.g., Abrines et al. 2012b; Gunnar et al. 2007; Hoksbergen et al. 2003; Kumsta et al. 2015).

Contrary to our expectations, time in institutional care, early deprivation, and prenatal alcohol exposure were not associated with ADHD symptom levels among Polish adoptees. This result is in contrast with previous research indicating that Romanian adoptees who had spent more time in institutional care showed more ADHD symptoms than children who had stayed less time in these institutions (Kreppner et al. 2001; Rutter et al. 2007). This finding was unexpected as well, given that increased ADHD symptoms have been observed among non-adopted children with a history of early deprivation (Maguire et al. 2015) and among children that were prenatally exposed to alcohol (Fryer et al. 2007). Yet, our results are in line with those of a previous study that examined more general behaviour problems in Polish adoptees (Knuiman et al. 2014).

Several explanations are possible for the lack of an association between ADHD symptoms and pre-adoptive risk factors in the current study. First, Polish adoptees had been exposed to multiple biological, prenatal, and early life risk factors prior to adoption (Knuiman et al. 2014). Other pre-adoptive risk factors were observed in Polish adoptees as well, such as parental abuse and parental mental disorders. As risk factors tend to co-occur, the co- occurrence of multiple risk factors might have obscured the individual contribution of institutional care, early deprivation, and prenatal alcohol exposure to ADHD symptoms in the current study. An alternative explanation for the lack of an association between ADHD symptoms and pre-adoptive risk factors might be the reliance on indirect information to measure children’s exposure to pre-adoptive risk factors. As we could not verify the information that adoptive parents provided on their children’s pre-adoptive experiences, it is unknown to what extent the information about pre-adoptive risk factors was accurate. Finally, the lacking association between ADHD symptoms and pre-adoptive risk factors might be due to the limited variability in our sample regarding time in institutional care. That is, the majority of the children had spent more than 6 months in institutional care (74%). Previous research showed that Romanian adoptees that had stayed more than 6 months in institutional care had more behaviour problems than children that had stayed less time in these institutions; there was, however, no additional effect of duration of institutionalization beyond the six- month cut-off (Kreppner et al. 2007).

The second aim of this study was to compare the association patterns and gender distribution of ADHD symptoms in Polish adoptees to those reported for ADHD symptoms in research among non-adopted children. As expected, ADHD symptoms were more strongly associated with attachment problems in Polish adoptees than these symptoms are in non- adopted children. This finding is consistent with those of previous studies reporting that adoptees with ADHD symptoms displayed increased attachment problems (e.g., Abrines et al. 2012b; Rutter et al. 2007; Stevens et al. 2008). Moreover, ADHD symptoms were more strongly associated with executive functioning deficits and less strongly with conduct problems in Polish adoptees than these symptoms are in non-adopted children. This finding is in agreement with previous research indicating that Romanian adoptees with increased ADHD symptoms displayed more impairment in executive functioning and less conduct problems, compared to non-adopted children with ADHD (Sonuga-Barke and Rubia 2008). Finally, our results revealed that ADHD symptoms in Polish adoptees were more equally distributed among boys and girls than they usually are in non-adopted children. This finding is in line with earlier studies reporting that ADHD symptoms in international adoptees equally affected boys and girls (e.g., Abrines et al. 2012b; Kreppner et al. 2001; Kumsta et al. 2015).

These findings are supportive of earlier suggestions that the association patterns of ADHD symptoms in internationally adopted children might be different from those of ADHD symptoms in non-adopted children (e.g., Kreppner et al. 2001; Kumsta et al. 2015). These differences might be due to a different underlying pattern of causation of attention and hyperactivity problems in international adoptees: It has been suggested that ADHD symptoms in these adoptees are linked to early environmental experiences, whereas ADHD symptoms in non-adopted children are considered to be strongly influenced by genetic factors (Taylor and Rogers 2005). This would be reflected in a higher risk for attachment problems and executive functioning deficits, and a lower risk for severe behaviour problems, like conduct problems, together with an absence of gender differences (Sonuga-Barke and Rubia 2008; Stevens et al. 2008). The patterns of associations found in the current study support this idea, pointing to an atypical presentation of ADHD symptoms in international adoptees. However, more research into the aetiology of ADHD symptoms in international adoptees is recommended, for instance at the underlying pathophysiological level, to better understand the dissimilarities to ADHD symptoms in the general population of children.

Thus, our findings are inconclusive with respect to theory about ADHD symptoms in international adoptees. On the one hand, the finding that ADHD symptoms were not associated with pre-adoptive risk factors opposes the suggestion that ADHD symptoms in international adoptees would constitute a specific response to early institutional deprivation (Rutter et al. 2001). This lack of an association, however, might be explained by characteristics of the current sample (e.g., co-occurrence of multiple pre-adoptive risk factors) or by methodological factors (e.g., indirect measurement of pre-adoptive risk factors). On the other hand, the finding that ADHD symptoms in Polish adoptees showed different association patterns than usually seen in non-adopted children supports the notion that ADHD symptoms in international adoptees would display atypical association patterns, possibly related to a different pattern of causation (e.g., Kreppner et al. 2001; Kumsta et al. 2015). Hence, this study strengthens the idea that international adoptees show increased ADHD symptoms with atypical association patterns; however, it is still unclear which factors or processes contribute to this different presentation of ADHD symptoms.

### Strengths, Limitations, and Future Research

This study expanded previous work by focusing on ADHD symptoms in children adopted from Poland. The use of Bayesian statistics enabled us to overcome the statistical limitations related to small sample size often encountered in previous adoption studies (e.g., Groza et al. 2008; Knuiman et al. 2014; Sonuga-Barke and Rubia 2008). We could use the data to directly evaluate multiple hypotheses of interest, which enabled us to obtain meaningful results despite the relatively small sample size (Van de Schoot et al. 2014).

Additionally, we were able to include previous empirical findings in our informative hypotheses (Kluytmans et al. 2012). By including background knowledge in the analyses, we could build upon previous research and update knowledge about ADHD symptoms in internationally adopted children. This updated knowledge may serve as background knowledge for other researchers in the field of international adoption as well.

There were, however, some limitations to this study. First, we only considered the perspective of the adoptive parents to measure children’s functioning after adoption. Our sole reliance on parental report might have inflated the associations between the different behavioural measures, due to same-reporter variance. Future studies that include additional informants or methods, such as teacher reports or observations, may provide more reliable associations and a more comprehensive picture of children’s functioning. Second, the use of two less validated questionnaires might be seen as a limitation of the current study. Although the BRIEF is the most widely used parental rating of executive functioning, low correlations have been reported between this instrument and experimental tasks on executive functioning (e.g., Bodnar et al. 2007). It has been suggested that the BRIEF may assess the role of executive functioning in daily life, rather than executive functioning at a fine-grained functional level (Huizinga and Smidts 2011). This should be taken into account when interpreting our findings concerning executive functioning deficits. Likewise, although the GIH mainly assesses attachment insecurity, some items might rather measure disinhibited social engagement. The GIH could therefore only be used as a general indication of attachment problems. We recommend that future studies use a measure that is better able to distinguish the different aspects of attachment problems. Third, we relied on retrospective information to assess children’s exposure to pre-adoptive risk factors. Therefore, we could not verify these pre-adoptive risk factors and some pre-adoptive circumstances were unknown to adoptive parents. It is recommended that future studies use additional sources of data, such as hospital records of the specific teratogens to which the children were prenatally exposed, to validate pre-adoptive risk factors. A fourth limitation relates to the formulation of informative hypotheses. Few studies were available that explicitly reported the parameters in which we were interested and that met our selection criteria. Therefore, some parameters were derived from studies with different samples or methodologies than used in the current study.

However, many of our hypotheses received much more support than the alternative hypotheses. We therefore expect that small differences in the parameters that were included in our informative hypotheses would have yielded the same conclusions, based on our analyses.

Furthermore, longitudinal studies on the functioning of Polish adoptees in adolescence and adulthood are recommended. It would be beneficial to examine how ADHD symptoms in this group of adoptees develop over time, together with their associated problems, such as attachment problems and executive functioning deficits. As longitudinal studies indicated that ADHD symptoms in Romanian adoptees persisted into early adolescence (Kreppner et al. 2007; Stevens et al. 2008), it might be expected that these symptoms are also persistent over time in Polish adoptees. Finally, it would be interesting to compare children adopted from Poland to children adopted from other countries, with respect to ADHD symptoms as well as their relationship to pre-adoptive risk factors and associated problems.

### Practical Implications

The high rates of clinically significant ADHD symptoms indicate that Polish adoptees and their adoptive parents may benefit from professional care after adoption. Adoptive parents should receive support after their child arrives in the family. Providing assistance to these parents at an early stage may prevent that the ADHD symptoms of their children will aggravate and impair their functioning in academic or social domains. Long-term assistance could be achieved by monitoring the development of Polish adoptees and providing help to adoptive parents when needed.

The atypical presentation of ADHD symptoms in this sample of Polish adoptees, in terms of association patterns and gender distribution, raises clinical issues. An important question is whether international adoptees with ADHD need a different clinical treatment than typically recommended for ADHD in non-adoptees (i.e., psycho-stimulant medication and psychosocial therapy; Wolraich 2012). It is unknown whether the standard pharmacological interventions will be effective in decreasing ADHD symptoms in adopted children, given their increased impairment in executive functioning, compared with non-adopted children with ADHD. On the other hand, the decreased association with conduct problems might facilitate treatment outcomes, since children with ADHD were found to benefit more from treatment than children with both ADHD and conduct problems (Ollendick et al. 2008). Research assessing the effectiveness of the standard ADHD treatments in adopted children with high levels of inattention, hyperactivity, and impulsivity is required.

Finally, the findings of this study will primarily apply to children adopted from Poland within the same time period. However, since the pre-adoptive backgrounds of CEE adoptees partially overlap, for instance regarding institutionalization (e.g., Miller et al. 2009), our findings may also be important to families and professionals working with children adopted from other CEE countries.

## Conclusion

Children adopted from Poland were at higher risk for displaying attention, hyperactivity, and impulsivity problems than Dutch children in the general population. Their ADHD symptoms showed atypical association patterns, which might indicate a different underlying causal mechanism of ADHD symptoms in international adoptees. Moreover, the majority of the Polish adoptees had experienced institutionalization, early deprivation, or prenatal alcohol exposure prior to adoption. These findings demonstrate that Polish adoptees and their adoptive parents need special attention, so that these children can develop in an optimal way.

## Footnotes

1 Posterior βs refer to the Bayesian equivalents of the standardized regression coefficient and posterior *SD*s to the Bayesian equivalent of the standard deviation. Posterior estimations are based on a compromise between prior information and the current data.
